# Epigenetic orchestration of RNA m^6^A methylation in wound healing and post-wound events

**DOI:** 10.7150/ijbs.114988

**Published:** 2025-07-28

**Authors:** Heao Zhang, Delong Gao, Zixin Li, Sis Aghayants, Yiping Wu, Zeming Liu, Qi Zhang

**Affiliations:** 1 Department of Plastic and Cosmetic Surgery, Tongji Hospital, Tongji Medical College, Huazhong University of Science and Technology, Wuhan, 430030, China.; 2 Department of Orthopaedic Surgery, Tongji Hospital, Tongji Medical College, Huazhong University of Science and Technology, Wuhan, 430030, China.; 3 College of Pharmacy, Jinan University, Guangzhou, 510632, China.; 4 Department of Plastic Surgery, Renmin Hospital of Wuhan University, Wuhan, Hubei, China.

**Keywords:** Wound healing, Post-wound events, Epigenetic modification, N^6^-methyladenosine, Scar, Skin appendage

## Abstract

Skin, the largest human organ, demonstrates remarkable regenerative capacity through spatiotemporally coordinated healing processes. Chronic wounds, including diabetic ulcers and burn injuries pose significant clinical challenges due to persistent inflammation, impaired angiogenesis, defective appendage regeneration, and pathological scarring. Emerging evidence reveals N^6^-methyladenosine (m^6^A) methylation - the most prevalent RNA modification - as a critical regulator of wound healing and tissue remodeling. The m^6^A machinery (writers, readers, erasers) dynamically controls RNA stability, translation, and splicing, thereby modulating keratinocyte migration, fibroblast activation, macrophage polarization, and stem cell differentiation. Dysregulated m^6^A dynamics impair diabetic wound healing through oxidative stress amplification and autophagy deficiency, while disrupting critical repair pathways in burn injuries. Aberrant m^6^A modifications exacerbate pathological scarring and dysfunctional appendage regeneration via dysregulated extracellular matrix deposition and fibroblast dysfunction. Current understanding of m^6^A spatiotemporal regulation and clinical potential remains fragmented despite significant advances. Future investigations integrating single-cell sequencing, spatial transcriptomics, and multidisciplinary approaches are crucial to decode the multifaceted roles of m^6^A, enabling the development of novel epitranscriptome-targeted therapies for chronic wound management and functional skin regeneration. The review systematically examines m^6^A-mediated mechanisms in cutaneous repair and remodeling, providing strategic insights for advancing regenerative medicine.

## Introduction

The skin is the largest organ of the human body, performing indispensable functions as a protective barrier 1. The skin consists of three primary layers, the epidermis, dermis, and hypodermis, which collectively enable sensory perception, provide physical, chemical, and biological defenses, maintain water-electrolyte balance, and thermoregulation 2. Chronic wounds, particularly those associated with diabetes, impose a significant burden on healthcare systems and society 3,4. Moreover, aberrant healing processes often compromise the structural and functional integrity of the skin, leading to diminished quality of life and presenting patients with both functional impairments and aesthetic challenges 5,6.

**Figure 1:**
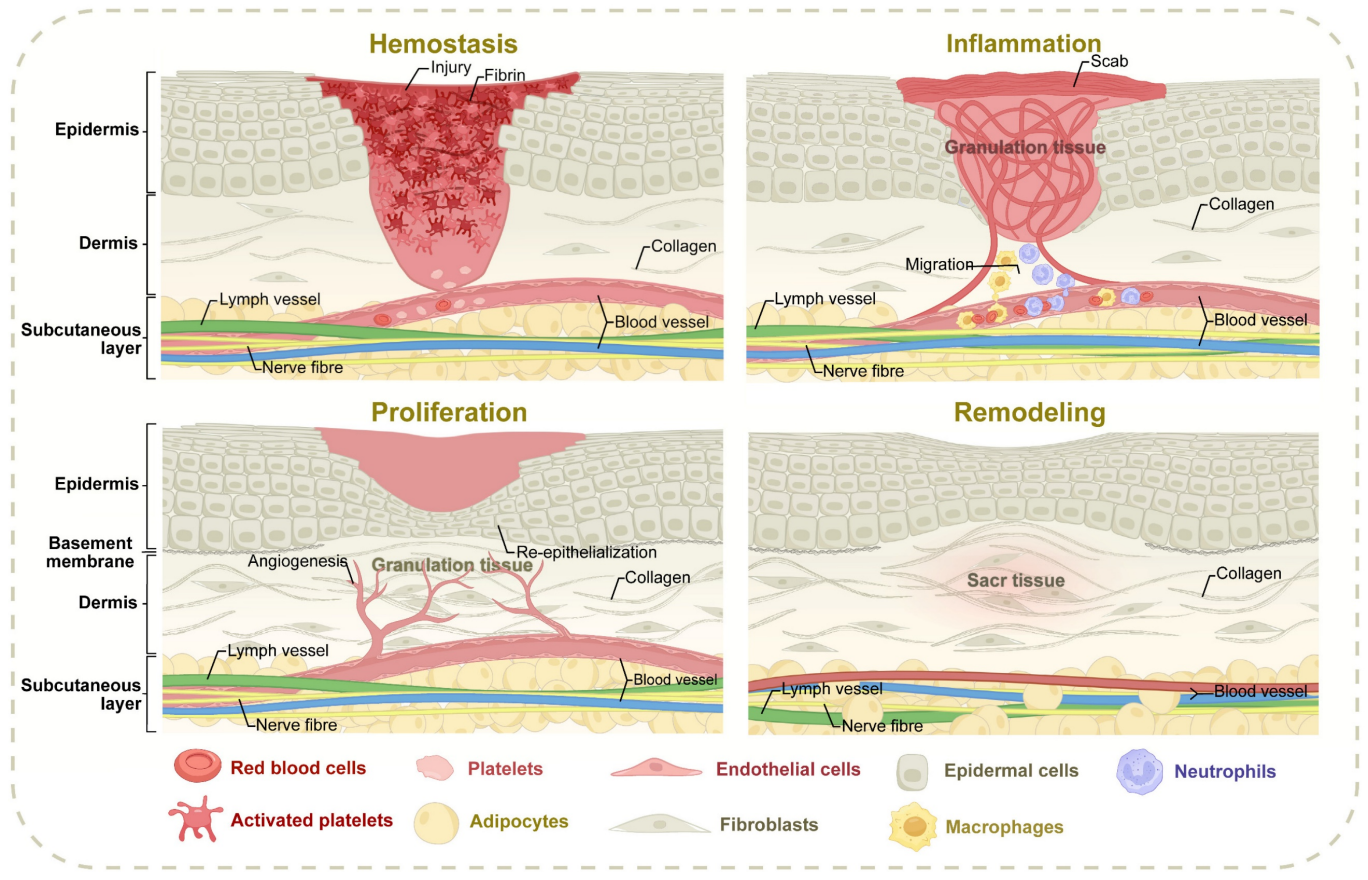
** The spatiotemporally coordinated processes of wound healing.** Normal wound healing progresses through four spatiotemporally coordinated phases: hemostasis (platelet aggregation/fibrin clot formation), inflammation (macrophage-mediated debris clearance and phenotypic switching), proliferation (angiogenesis, re-epithelialization, and fibroblast-driven granulation tissue formation), and remodeling (ECM maturation via collagen I/III dynamics and vascular pruning). The abnormal healing processes may arise from specific microenvironmental imbalances encompassing macrophage polarization defects in diabetic conditions, pathological fibroblast activation associated with hypertrophic scars, epidermal progenitor cell functional impairment within skin appendages, and oxidative stress cascades triggered by sustained hyperglycemia, all of which collectively disrupt the spatiotemporal coordination of healing phases and diminish the structural-functional restoration of damaged tissues.

Wound healing is a complicated biological process aimed at restoring skin integrity and can be categorized into four distinct and sequential phases: hemostasis, inflammation, proliferation, and remodeling 7 **(Figure 1)**. During the hemostasis phase, vasoconstriction initially minimizes microvascular hemorrhage 8. Exposed collagen within the wound promotes platelet chemotaxis and aggregation, which, in conjunction with the insoluble fibrin network formed by the coagulation cascade, establishes a thrombus to seal the injury site 9,10. The inflammation phase is characterized by the recruitment of inflammatory cells to the wound under the regulation of intricate signaling pathways. These cells work to clear necrotic tissue and prevent infection, creating a foundation for subsequent phases 11. Among them, macrophages can remove extracellular matrix (ECM) and cell debris, and their dynamic transformation from pro-inflammatory to anti-inflammatory phenotype is crucial to the evolution of the healing process. Macrophages, in particular, are critical in removing ECM components and cellular debris, with their dynamic transition from pro-inflammatory to anti-inflammatory phenotype being essential for healing progression 12-14. The proliferation phase involves the simultaneous and coordinated regeneration of various skin components, including angiogenesis, re-epithelialization, lymphangiogenesis, and the regeneration of hair follicles and appendages 15. Fibroblasts proliferate to form granulation tissue, with some differentiating into myofibroblasts to contract wound edges 16. In the remodeling phase, fibroblast apoptosis and macrophage-mediated degradation of fibrous components drive the dynamic restructuring of the ECM 17. This stage involves ECM transformation, cyclic deposition, and pruning of newly formed vasculature 18. Granulation tissue undergoes structural reorganization to support tissue maturation, a process that can span several years to ensure the stability and functionality of the repaired tissue 19. The progression of wound healing is influenced by numerous factors, including the underlying causes of the wound and individual patient variability 20,21. Disruptions to normal healing events can result in atypical outcomes, with the final stages often failing to fully restore the original morphology and functionality of the tissue. Understanding these deviations is critical for addressing impaired healing and developing targeted therapeutic strategies tailored to specific wound etiologies and patient conditions.

Patients with diabetes often experience chronic low-grade inflammation driven by persistent hyperglycemia, which severely compromises wound healing and predisposes them to the formation of chronic wounds 22. During the inflammation phase of wound healing, the phenotypic transition of macrophages to the M2 subtype is notably impaired in diabetic wounds, consequently diminishing the rate of wound resolution and heightening susceptibility to infections 23. Hyperglycemia, combined with the insufficient presence of M2 macrophages, further inhibits the proliferation of critical regeneration-related cell types, including endothelial cells, keratinocytes, and fibroblasts 24,25. Compared with normal wound environments, diabetic wounds are exacerbated by concomitant peripheral vascular insufficiency and neuropathy 26, and these complications aggravate the wound microenvironment and delay re-epithelialization, thereby perpetuating the state of chronic wound pathology. Burns are defined as injuries caused by direct contact with heat sources 27,28. The wound surface in burn injuries could be subdivided into three distinct zones based on the proximity to the heat source, alterations in blood flow, and the extent of tissue damage 29. The zone of coagulation is the most critical area, characterized by irreversible tissue necrosis due to extreme temperature elevation and protein denaturation 30. Adjacent to this is the zone of stasis, which is predominantly marked by ischemia, hypoxia, and hypoperfusion. However, with timely and appropriate intervention, tissue damage in this zone could be reversible. The outermost layer, the zone of hyperemia, demonstrates enhanced tissue recovery capacity, attributable to the inflammatory reflex-mediated vasodilation 30. Burn injuries provoke a cascade of physiological disruptions, such as exaggerated inflammatory responses, distributive shock, and increased susceptibility to infections. These factors collectively hinder the healing process by delaying wound closure and promoting scar formation 31-33.

During the proliferation and remodeling phases of wound healing, excessive cellular proliferation or abnormal ECM deposition can lead to hypertrophic scars (HSs) and keloids 34. Such scarring typically arises from wounds penetrating beyond the dermis, characterized by excessive fibroblast proliferation 35. In the proliferation phase, prolonged inflammatory stimulation activates fibroblasts and sustains myofibroblast populations, driving excessive Type III collagen deposition 36,37. During the remodeling phase, the replacement of Type III collagen with Type I collagen ultimately contributes to scar formation 38.

Skin appendages, including hair follicles, sebaceous glands, and sweat glands, are capable of maintaining skin homeostasis, wound healing, and tissue regeneration. Sebaceous glands secrete lipids that help maintain skin hydration and barrier functions, while hair follicles provide physical protection and aid in the distribution of sweat and sebum 39,40. During wound healing, sebaceous gland and hair follicle progenitor cells migrate to injury sites, promoting re-epithelialization and restoring epidermal integrity 41. Eccrine sweat glands assist in thermoregulation and metabolic waste removal, and sweat ductal basal progenitors rebuild sweat gland structures during healing 42. Enhancing skin appendage regeneration and reducing HS contributes to scarless wound healing and restores physiological function of the skin.

Epigenetics generally refers to heritable changes in gene expression that do not alter the underlying DNA sequence 43. These changes include DNA methylation, histone modifications, and non-coding RNA (ncRNA), which together regulate chromatin structure and gene function 44,45. With the advent of RNA epitranscriptomics, RNA modifications have become crucial in various physiological and pathological processes through chemical alterations on ribose and nucleobases 46,47. Among over 170 known types of RNA modifications, RNA methylation is predominant, with key forms including 3-methylcytidine (m^3^C), N^7^-methylguanosine (m^7^G), N^6^-methyladenosine (m^6^A), 5-methylcytosine (m^5^C), and N^1^-methyladenosine (m^1^A) 48. These modifications occur across diverse RNA species, such as long non-coding RNA (lncRNA), messenger RNA (mRNA), transfer RNA (tRNA), and ribosomal RNA (rRNA), regulating RNA stability, translation, splicing, degradation, and transport 46. The m^6^A modification involves methylation at the N^6^ position of adenosine, primarily found in mRNA and lncRNA 49. As the most prevalent RNA modification in eukaryotes, it is typically enriched at DRACH motifs (D = A/G/U; R = A/G; H = A/C/U) within internal long exons, 3' untranslated regions (3'-UTRs), and sequences adjacent to stop codons, contributing to the stability of RNAs 50. On average, each mRNA contains 3-5 m^6^A sites 43,51.

The m^6^A modification is reversibly and dynamically regulated by methyltransferase (m^6^A writers) such as METTL3/METTL14 complexes, demethylases like ALKBH5 and FTO (m^6^A erasers), and m^6^A-binding proteins (m^6^A readers). These components are indispensable for RNA metabolic processes 52-54. The detection of m^6^A modifications has advanced considerably, revealing their dynamic regulation in response to environmental changes 55,56. Initial methods like MeRIP-seq and m^6^A-seq used m^6^A-specific antibodies but had limited resolution. Advanced techniques, including enzymatic labeling techniques such as DART-seq, m^6^A-label-seq, m^6^A-SAC-seq, and m^6^A-REF-seq, along with single-base-resolution and quantitative methods like GLORI and eTAM-seq, have greatly enhanced mapping precision 57. However, critical challenges regarding RNA integrity and accuracy constraints in low-abundance transcripts remain incompletely resolved. Furthermore, integration with single-cell sequencing, machine learning, and third-generation sequencing technologies further refines m^6^A detection 58.

Previous studies have extensively investigated the mechanisms of m^6^A modifications in diverse biological processes, such as tumorigenesis, embryonic development, and the central and hematopoietic systems 59,60. Recent studies showed that m^6^A modification also dynamically regulated autophagy and inflammatory responses of the skin during wound healing 61. In inflammation models, the downregulation of METTL3 and METTL14 promoted inflammation activation, and METTL3 upregulation drove M1 macrophage polarization 62-64. Additionally, m^6^A epigenetically can modulate key cellular processes during wound healing, such as proliferation, differentiation, migration, angiogenesis, re-epithelialization, and the formation of skin appendages, ultimately influencing healing outcomes 65,66. Accordingly, this review aims to systematically elucidate the critical roles and mechanisms of m^6^A RNA methylation in skin wound healing and post-wound events. We highlight the potential of m^6^A as a therapeutic target for improving outcomes in diabetic wounds, burn injuries, scar formation, and skin appendages.

## The m^6^A Regulators

### The m^6^A writers

The m^6^A writers function as RNA methyltransferases that catalyze the site-specific N6-methylation of adenosine in RNAs, adding m^6^A marks to RNA 67. The core writer complex consists of METTL3 and METTL14, which form a 1:1 heterodimeric structure indispensable for m^6^A methylation 68. METTL3 contains a catalytic domain critical for transferring the methyl group from S-adenosylmethionine to the ribose of adenosine residues 69. METTL14, while lacking catalytic activity, stabilizes the complex and enhances the substrate specificity by recognizing RNA motifs 70. Accessory proteins such as WTAP and ZC3H13 further modulate the enzymatic activity and subcellular localization of the METTL3-METTL14 complex, ensuring precise m^6^A deposition at specific genomic sites 71. Recent advances in this field have expanded the repertoire of m^6^A writers to include METTL5, METTL7A, METTL7B, METTL16, and ZCCHC4 72,73. All these writers collectively regulate m^6^A deposition dynamically, orchestrating diverse post-transcriptional networks and influencing various cellular processes such as gene expression, RNA stability, and translation efficiency.

### The m^6^A readers

The m^6^A readers are proteins equipped with specific domains that recognize and bind m^6^A-modified RNA, influencing downstream biological processes. YTH domain-containing proteins such as YTHDF1, YTHDF2, and YTHDF3 represent a major family of readers that adopt β-propeller folds, enabling high-affinity binding to m^6^A residues 74. These readers can regulate RNA stability, splicing, and translation by recruiting auxiliary factors or directly interacting with RNAs. For instance, YTHDF2 promotes RNA degradation by recruiting the CCR4-NOT deadenylase complex, and YTHDF1 enhances translation by interacting with ribosomal subunit 73. Beyond YTH proteins, other reader families, such as HNRNPs and IGF2BPs, utilize distinct structural motifs to recognize m^6^A, expanding the diversity of m^6^A-mediated regulatory mechanisms 75.

### The m^6^A erasers

The m^6^A erasers, primarily ALKBH5 and FTO, are RNA demethylases that enzymatically reverse m^6^A modifications by removing methyl groups from adenosine residues 76. Structural studies of ALKBH5 reveal an α-KG-binding site and a catalytic cavity where the m^6^A residue is accommodated for demethylation 77. The reaction involves the abstraction of a proton from the methyl group, followed by oxidative cleavage to restore the unmodified adenosine 78. FTO exhibits a similar catalytic mechanism but shows distinct substrate preferences and tissue-specific expression patterns 79. Beyond their intrinsic enzymatic activity, the m^6^A erasers also interact with RNA-binding proteins to form complexes that regulate RNA metabolism. These erasers play pivotal roles in dynamically modulating the m^6^A landscape, ensuring precise control of RNA stability, splicing, and translation in response to cellular and environmental changes 80.

### The dynamic regulatory role of m^6^A in RNA metabolism

**Figure 2:**
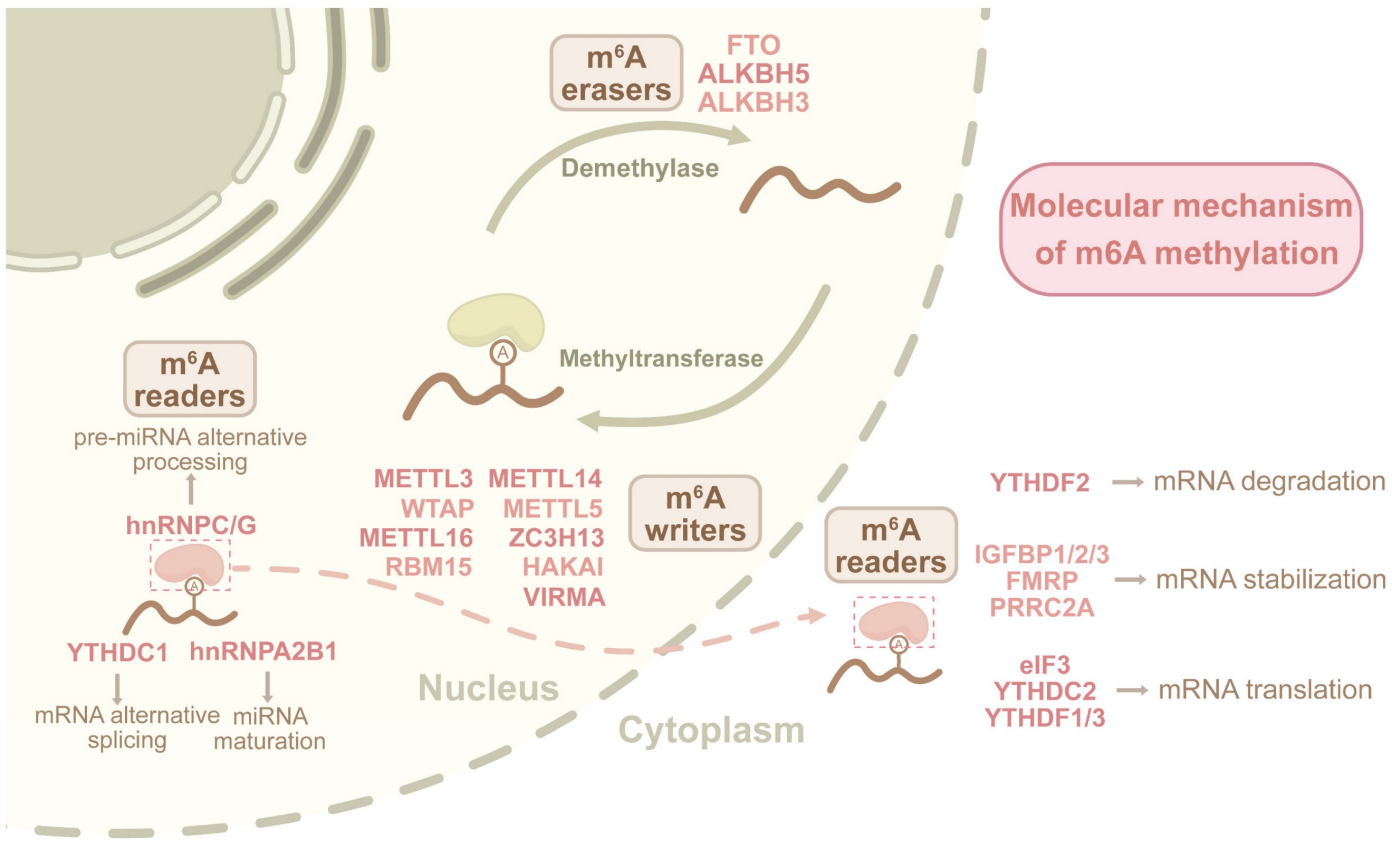
** Dynamic m^6^A regulatory mechanism in RNA metabolism.**The m^6^A modification is orchestrated by writers (METTL3/14 complex with accessory proteins), readers (YTHDFs, HNRNPs) recognizing methylated adenosines, and erasers (FTO/ALKBH5) mediating demethylation. Writers establish m^6^A marks through catalytic methylation, readers decode these marks to regulate RNA stability, splicing, and translation, while erasers dynamically erase modifications. This tripartite system governs spatiotemporal control of RNA processing, influencing cellular metabolism and function.

The m^6^A modification is catalyzed by the methyltransferase complex, primarily composed of METTL3, METTL14, and WTAP, which add methyl groups to specific adenosine residues in RNAs 81. This modification is subsequently recognized by m^6^A readers, modulating RNA stability, alternative splicing, nuclear export, and translational efficiency, thereby influencing cellular differentiation, tumorigenesis, and immune regulation 82. The removal of m^6^A is mediated by m^6^A erasers FTO and ALKBH5, which reverse methylation to destabilize mRNA or alter its translational potential, thereby impacting stem cell fate, DNA damage response, and metabolic reprogramming in cancer 83 **(Figure 2)**. The m^6^A writers, readers, and erasers form a collaborative network that balances RNA modifications, ultimately regulating RNA fate and cellular responses.

## The m^6^A modification in normal wound

### Atlas of m^6^A in normal wound

The m^6^A modification plays a crucial regulatory role in biological processes like tissue morphogenesis and cellular differentiation. Recent findings reveal its novel impact on wound healing by modulating keratinocyte, fibroblast activity and other cell fates.

### m^6^A writers in normal wound

The lncRNAs lack protein-coding capacity, but could critically impact cellular metabolism, migration, and proliferation by regulating gene transcription and translation 84-86. The dynamic regulation of m^6^A could interact with lncRNA to intricately govern stem cell differentiation pathways during skin development 87. The plasmacytoma variant translocation gene 1 (PVT1) encodes an lncRNA located on chromosome 8 in the 8q24 region, which is a recognized cancer risk region and is adjacent to the myelocytomatosis oncogene (MYC). Recent studies further identified its regulation of epidermal differentiation as well as wound healing 88. Lee et al. showed that the conditional knockout of m^6^A writer METTL14 in mice resulted in impaired skin development and inhibited wound healing 89. METTL14 could modify lncRNA PVT1 to stabilize MYC, thus increasing p63^+^ basal cell number, epidermal thickness, and spinous layer, as well as maintaining the stemness of epidermal progenitor cells. The activation of the METTL14-PVT1 axis significantly enhanced the stemness of epidermal progenitor cells, thereby accelerating wound healing.

### m^6^A readers in normal wound

The IGF2BPs constitute a distinctive group of m^6^A readers that interact with the GG(m^6^A)C consensus motif, facilitating the stabilization and retention of target mRNAs in an m^6^A-dependent manner 90. By bioinformatics analysis, Zhi et al. found that IGF2BP2 and IGF2BP1 expressions were upregulated in human skin wounds 91. And, IGF2BP2 could enhance the stability of heparanase by binding to 3'-UTR of heparanase. This led to the promoted proliferation, migration, and angiogenesis of HaCaT cells *in vitro*, implicating IGF2BP2 as a vascular-associated target of wound healing. Wu et al. discovered that IGF2BP2 expression was upregulated in the wound margin epidermis of wild-type mice, while overexpressed microRNA (miRNA) let-7b could suppress the IGF2BP2 expression in HaCaT cells by targeting 3'-UTR, thus inhibiting cell migration *in vitro* 92. In addition, let-7b downregulated re-epithelialization and wound healing processes in mice through the let-7b-IGF2BP2 axis, thereby repressing the wound healing process. This study emphasized the specific capability of let-7b in impacting wound healing by targeting IGF2BP2.

### m^6^A erasers in normal wound

The ALKBH5 enhanced neuronal survival and plasticity through m^6^A demethylation, while hindering axonal regeneration in both the central and peripheral nervous systems 93,94. In immune regulation, ALKBH5 promotes CD4^+^ T cell activity and neutrophil migration under inflammatory conditions and supports antimicrobial responses 95,96. In the wound skin in mice, Huang et al. demonstrated that the overall m^6^A modification was decreased with ALKBH5 and FTO upregulation 97. The ALKBH5 promoted migration of HaCaT cells but with little alteration of cell apoptosis or proliferation. Furthermore, ALKBH5, along with m^6^A reader YTHDF2, promoted PELI2 expression by removing the m^6^A modification and enhanced the stability of PELI2 both in HaCaT cells and mice, thus contributing to keratinocyte migration, re-epithelialization, and skin wound healing. Removal of the m^6^A modification of PELI2 by ALKBH5 offered an m^6^A-targeted conception for re-epithelialization in wound healing.

## The m^6^A Modification in Diabetic Wound

### Atlas of m^6^A in diabetic wound

While normal wound repair progresses through tightly coordinated phases of inflammation resolution and tissue regeneration, diabetic wounds exhibit a prolonged healing trajectory under hyperglycemic stress, which is exacerbated by persistent chronic inflammation, suppressed autophagic activity, and the accumulation of advanced glycation end products (AGEs). The m^6^A modification maintains RNA homeostasis in physiological conditions but becomes functionally compromised under metabolic stress 61, and further investigation is warranted to clarify the dynamic changes of m^6^A within these critical processes. Shen et al. reported that the global m^6^A levels were decreased in MeRIP-Seq of mice diabetic wound tissue, with 777 downregulated peaks out of 1335 significantly different m^6^A peaks 98. For m^6^A regulators, ALKBH5 was upregulated while METTL3, METTL14, and WTAP were downregulated in diabetic wounds. In addition, the m^6^A upregulated peaks were enriched in immune response, metabolic process, and redox reaction, indicating possible physiological functions regulated by m^6^A.

### m^6^A writers in diabetic wound

The human umbilical cord mesenchymal stem cells (hUCMSCs) mitigated oxidative stress-induced damage in HUVECs through exosome-mediated secretion, which concurrently reduces the inflammatory response. Furthermore, exosomes derived from hUCMSCs could promote blood perfusion, angiogenesis, and granulation tissue formation, thereby facilitating the acceleration of diabetic wound healing 99. Huang et al. demonstrated that lncCCKAR-5 was downregulated in hUCMSCs treated with high glucose, which intensified the autophagic effect 100. However, the m^6^A writer METTL3 could modify lncCCKAR-5 and enhance the formation of lncCCKAR-5/LMNA/MKRN2 complex in hUCMSCs. This complex then promoted expressions of senescence-related genes and ubiquitin-mediated degradation of LMNA, and inhibited the autophagy process, leading to the impaired healing function of hUCMSCs in diabetic wounds in nude mice.

### m^6^A readers in diabetic wound

Elevated glucose levels in diabetic wounds disrupt autophagy, hindering cellular migration and re-epithelialization, and this process is intricately regulated by m^6^A methylation 101 102. The m^6^A reader YTHDC1 regulates embryonic development by modulating mRNA splicing, polyadenylation, and chromatin accessibility 103. Liang et al. demonstrated that YTHDC1 exhibited decreased expression in HaCaT cells exposed to hyperglycemia, consequently leading to the downregulation of SQSTM1 and autophagy-related genes, accompanied by elevated apoptosis rates 104. Consistently, YTHDC1 downregulation in db/db mice accelerated the degradation of SQSTM1 mRNA and reduced autophagic flux, thus resulting in impaired wound healing, evidenced by thinner epidermis and delayed diabetic wound healing process.

Circular RNAs (circRNAs), characterized by their covalently closed-loop structures and resistance to degradation, have been shown to critically influence diabetic wound healing by impairing keratinocyte migration. Huang et al. discovered that m^6^A reader IGF2BP3 could interact with circCDK13 in an m^6^A-dependent manner to enhance the stability of each other 105. This promoted circCDK13 function in increasing CD44 and c-MYC expression, thus enhancing the proliferation and migration of human dermal fibroblasts (HDFs) and human epidermal keratinocytes (HEKs) *in vitro*. Furthermore, the injection of circCDK13-overexpressing small extracellular vesicles (sEVs) significantly upregulated the expression of IGF2BP3, then accelerated healing and skin regeneration of full-thickness cutaneous wounds in db/db mice with diabetes, which provided an m^6^A-circRNA axial therapy for diabetic wound healing.

### m^6^A writer-reader pairs in diabetic wound

The interaction between m^6^A readers and target RNAs alters the RNA half-life, thereby influencing the downstream effects of m^6^A modifications. This reader-dependent modification can either amplify or attenuate downstream consequences.

By examining clinical samples, Wang et al. found that METTL3 expression was reduced by more than 50% in the tissues of DFU patients compared with normal human tissues. Moreover, the degree of METTL3 reduction was positively correlated with DFU severity 106. The m^6^A modification by METTL3 and IGF2BP2 enhanced the stability, expression, and function of NDUFB5, thereby reversing the inhibition of cell viability, migration, and mitochondrial respiration of HUVECs induced by AGEs. Further exploration demonstrated that the expressions of NDUFB5 and METTL3 were downregulated in diabetic wounds of STZ-induced diabetic mice, while the overexpression of NDUFB5 accelerated diabetic wound healing in mice. This study confirmed the pivotal role of METTL3-mediated NDUFB5 m^6^A modification in accelerating DFU wound healing. Zhou et al. demonstrated that the overexpression of METTL3 in ADSCs accelerated the migration and proliferation of LECs in the co-culture model. Furthermore, m^6^A modification by METTL3 and IGF2BP2 in ADSCs elevated VEGF‑C expression and VEGF‑C‑mediated lymphangiogenesis in diabetic wounds of mice, therefore contributing to diabetic wound healing 107. Therefore, METTL3 functioned in enhancing lymphangiogenesis and improving ADSC efficiency in DFU treatment.

## The m^6^A modification in burn injury

### Atlas of m^6^A in burn injury

Elevated levels of cytokines and chemokines in burn wounds drive a pronounced inflammatory response, while intensified oxidative stress induces endothelial damage, capillary leakage, and distributive shock 108,109. Furthermore, adaptive immunity is markedly compromised, with reduced T cell proliferation and increased susceptibility to infections, compared to uninjured skin 110. Ran et al. discovered that the overall m^6^A levels in burned human skin were downregulated compared with normal skin, with hypomethylation observed in 754 lncRNAs and 5492 mRNAs 111. Functional enrichment results showed that hypermethylated mRNAs promoted inflammatory responses, while hypomethylated mRNAs inhibited healing-related pathways such as supramolecular fiber organization and protein catabolism. The downregulation of the m^6^A regulators, including NRNPC, FMR1, ALKBH5, METTL16, and METTL14, suppressed overall m^6^A levels and resulted in low mRNA expression, thus hindering physiological processes in wound healing.

### m^6^A modification in burn injury

**Figure 3:**
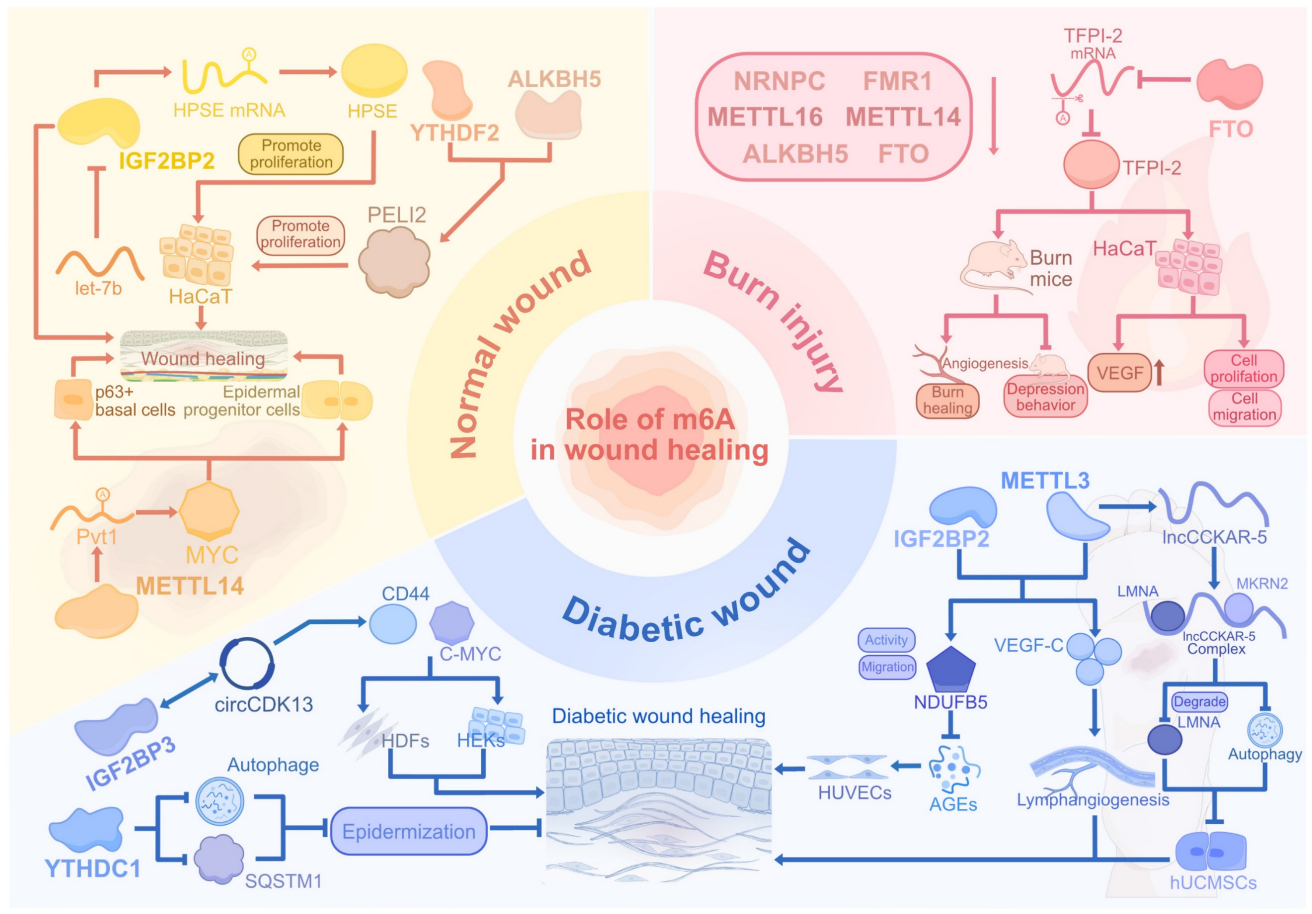
**The regulatory roles of m^6^A modification in normal, diabetic, and burn wounds.** The m^6^A dynamics orchestrate tissue repair by regulating keratinocyte and fibroblast migration, epidermal stemness, and angiogenesis through writers, readers, and erasers. In diabetic wounds, METTL3-IGF2BP2 enhances mitochondrial and lymphatic repair while hyperglycemia disrupts autophagy via YTHDC1-IGF2BP3. Burn injuries show FTO-mediated vascular restoration and METTL depletion-induced oxidative stress. Pathological conditions like hyperglycemia and thermal injury perturb m^6^A homeostasis, impairing re-epithelialization and amplifying inflammation through dysregulated RNA networks and senescence signals. Therapeutic targeting of m^6^A regulators, such as METTL3-IGF2BP2 and FTO, mitigated inflammation and oxidative damage, promoting repair across wound subtypes.

FTO regulates adipogenesis and fat metabolism through RNA demethylation and has been implicated in the progression of various cancers by promoting cell proliferation, migration, and metastasis 112-114. In keratinocytes, low-level arsenic exposure upregulates FTO expression, leading to a reduction in m^6^A methylation and the promotion of tumorigenic behaviors 115. Xu et al. found that m^6^A eraser FTO was downregulated in both heat-stimulated HaCaT cells and human burn skin 116. The overexpression of FTO inhibited TFPI-2 expression through m^6^A modification, which contributed to proliferation, migration, and VEGF expression in heat-stimulated HaCaT cells. In addition, the overexpression of FTO not only accelerated small blood vessel number, angiogenesis, and wound healing but also decreased depressive-like behaviors in the burn rat model. This suggests FTO as a potential m^6^A target for treating burn injuries and related complications** (Figure 3)**.

## The m^6^A modification in Scars

### Atlas of m^6^A in scars

Fibrosis within dermal tissue during wound healing can result in pathological scarring, which is commonly observed when skin injuries extend deep into the dermis 117,118. Throughout the proliferative phase, excessive scarring is facilitated by excessive deposition of ECM and sustained fibroblast recruitment, activation, and differentiation of fibroblasts. In the subsequent remodeling phase, impaired re-epithelialization further contributes to the development of excessive scarring. Excessive scarring manifests primarily as hypertrophic scars and keloids. Keloids, representing the more severe form, can extend beyond the original wound boundaries and do not regress. The keloid tissue also exhibits more disorganized deposition of type I and III hypocellular collagen bundles, and demonstrates a higher recurrence rate after surgical excision 119.

Recent advances in transcriptomic sequencing have revealed gene expression differences in scar tissues, highlighting the role of m^6^A methylation in scar formation. By merging two bulk RNA-seq databases, Yang et al. discovered that m^6^A writers METTL3 and METTL14 were downregulated while IGF2BP3 was upregulated in keloid samples 120. The expression levels of m^6^A writers and m^6^A erasers showed a positive correlation due to their negative feedback regulation, and the diagnosis model using the expressions of m^6^A regulators showed excellent predictive ability. Xie et al. identified hub genes strongly correlated with keloid development by analyzing bulk RNA-seq sequencing of human keloid tissue and keloid fibroblasts 121. Then they constructed a keloid diagnostic Lasso model based on 5 genes and divided keloid samples into high-risk and low-risk groups, showing that m^6^A regulators ALKBH5, FTO, and HNRNPA2B1 were upregulated in the high-risk group, while YTHDF2 was downregulated. Hence, alterations in m^6^A-related gene expression are closely linked to scar tissue development. In HS, m^6^A sequencing revealed a decrease in the original 7,835 m^6^A peaks and an increase in 14,791 new peaks 122. The genes associated with these m^6^A peaks were enriched in fibrosis-related pathways, and mRNA expression levels were positively correlated with m^6^A modifications. Notably, COL11A1, COL8A1, CYP1A1, and AR showed the highest expression and m^6^A levels in HTS. These findings provide a basis for further exploration of m^6^A-modified mRNA in HS.

### m^6^A writers in scars

The m^6^A modifications exert significant influence on tissue regeneration, wound healing, and fibrosis by modulating processes such as epithelial-mesenchymal transition (EMT), cell type differentiation, and regulating diverse signaling networks. Elevated m^6^A levels have been observed as a hallmark of keloid formation. Fibroblasts, along with critical fibrosis-associated factors and pathways such as TGF-β and the Wnt/β-catenin signaling axis, are crucial modulators in altering cellular functions induced by m^6^A modification.

By MeRIP sequencing, Lin et al. found the elevated m^6^A level in keloid tissue, with the m^6^A region mainly located in 3'-UTR and the methylation peaks mainly located on chromosomes 1, 17, and 19 123. Among all m^6^A regulators, the levels of METTL3 and WTAP were significantly higher in keloid samples, while the levels of ALKBH5, FTO, and METTL14 exhibited little differences. Additionally, the Wnt and TGF-β pathways in keloids contained numerous high m^6^A level genes, and the Wnt/β-catenin/S100A4 pathway was elevated in human keloid samples, confirming Wnt signaling activation by m^6^A modification in keloids. Liu et al. further reported that TGF‑β1 elevated the m^6^A level in human Tenon's capsule fibroblasts (HTFs) through the upregulation of m^6^A writers METTL3, METTL14, and WTAP 124. In addition, the upregulation of METTL3 could also promote TGF-β/Smad signaling to enhance viability, proliferation, and ECM deposition in both HTFs and rabbit models of Glaucoma filtration surgery (GFS). The downregulation of METTL3 expression and TGFβ/Smad3 signaling may improve the prognosis of glaucoma surgery. Additionally, activation of homeodomain-interacting protein kinase (HIPK2) has been shown to promote fibrosis in multiple organs through signaling pathways such as Wnt/β-catenin, TGF-β, and Notch Moreover, the overexpression of m^6^A writer ZC3H13 mediated the m^6^A modification of HIPK2 to maintain its stability, then promoted the proliferation and migration of human keloid fibroblasts (HKFs) but inhibited apoptosis *in vitro*, which led to keloid formation 125. Inhibiting the ZC3H13-HIPK2 axis might be beneficial for reversing fibrosis and keloid formation.

### m^6^A erasers in scars

The total m^6^A levels of keloids were decreased, and the overexpression of FTO in human fibroblasts enhanced cell migration 126. In addition, FTO overexpression stabilized collagen I α-chain (COL1A1) by reducing the m^6^A modification of COL1A1 and upregulated α-smooth muscle actin expression, which contributed to collagen deposition. Nevertheless, the FTO overexpression could be inhibited by glucocorticoids, and this provided a potential mechanism of glucocorticoid treatment in keloids via m^6^A-related pathways.

Similarly, ALKBH5 is an essential m^6^A demethylase and is involved in inhibiting the macrophage-to-myofibroblast transition induced by hypertensive stress and in curtailing hepatic stellate cell (HSC) proliferation, thereby functioning as a regulatory factor. The level of m^6^A modification was elevated in human keloid tissue, and the expression level of m^6^A eraser ALKBH5 was negatively correlated with the severity level of HSs 127. ALKBH5 deficiency led to increased m^6^A levels in downstream targets COL3A1, COL1A1, and ELN, then the m^6^A reader YTHDF1 bound to these three targets to stabilize and upregulate expression. This led to pathological deposition and the remodeling of ECM in both vivo and vitro, finally resulting in HS. However, the overexpression of ALKBH5 inhibited the expression of downstream targets in HSFs and mice, thereby inhibiting the contraction of HSFs. The ALKBH5-mediated m^6^A demethylation ameliorated ECM deposition in cutaneous pathological fibrosis, suggesting a potential option for reversing pathological fibrosis.

## The m^6^A Modification in Skin Appendages

### Atlas of m^6^A in skin appendages regeneration

In severe skin wounds, the loss of functional skin cells and the disruption of the skin mechanical barrier make wound repair challenging. During the healing process, the absence of hair follicle stem cells, impaired activation of epidermal stem cells, and scar formation further hinder the regeneration of skin appendages 128. The m^6^A modification is a crucial orchestrator in regulating cell apoptosis, development, and proliferation 55. It is essential for maintaining stem cell pluripotency and guiding differentiation 129,130. Therefore, during wound healing, exploring the relationship between m^6^A and the development and regeneration of skin appendages may help identify strategies to restore the structure and function of these appendages.

### m^6^A modification in hair follicle growth and regeneration

To initiate a new hair follicle growth process, promoting signals from dermal cells, such as FGF7 and TGFB2, along with BMP inhibiting signals, can be received by epidermal stem cells 131. The upregulation of various signals, including Wnt and Shh, subsequently creates an essential microenvironment for epidermal stem cell proliferation, differentiation, and ultimately hair formation 132,133. Xi et al. found that the m^6^A peaks in mouse epidermis were enriched in key pathways for hair follicle formation, including Wnt and Shh 134. METTL3-cKO newborn mice showed epidermal development disorders, with impaired WNT and SHH signaling pathways. Cellular experiments demonstrated that METTL3 knockout in epidermal progenitor cells led to a reduction in Wnt and Shh signaling pathways, and hair follicle formation, with an increase in sebaceous cell formation. López et al. also found the role of METTL3 in epithelial development and differentiation. Compared with WT mice, newborn METTL3-eKO mice exhibited abnormal epidermal development, including a thicker granular layer and stratum corneum, loss of basal cell polarity, hair follicle deformation, and ultimately a more differentiated cell phenotype 135. METTL3 Knockout in epidermal tissue cells increased the half-life and expression level of epigenetic modifier mRNA, which promoted the cell differentiation process. METTL3 is essential for proper hair follicle development and cellular differentiation through modulating Wnt and Shh signaling pathways.

### m^6^A modification in sweat gland growth and regeneration

**Figure 4:**
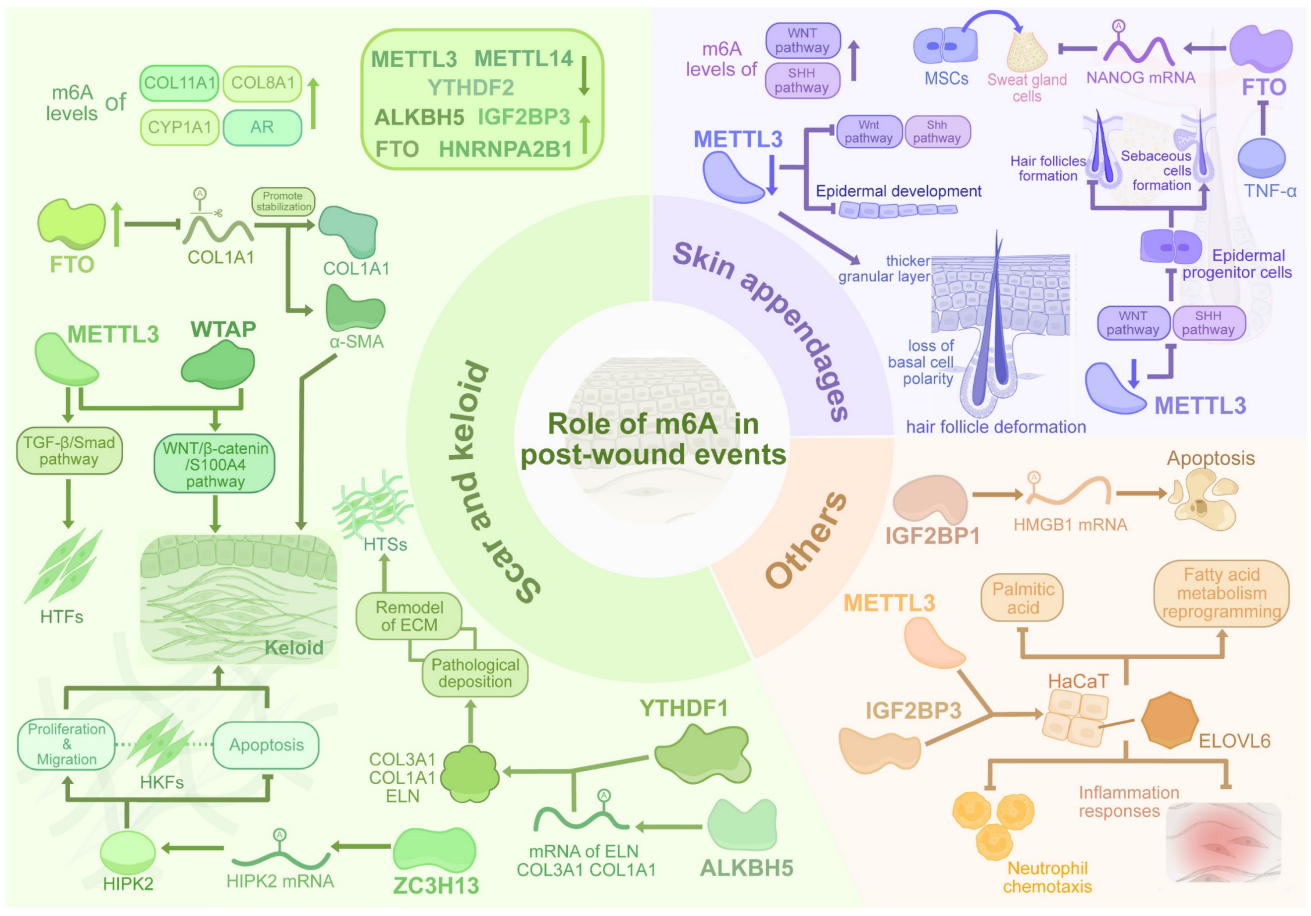
** The dynamic regulation of m^6^A methylation in pathological scarring, skin appendage regeneration, and other events.** Dysregulated m^6^A writers (METTL3, WTAP) and erasers (FTO, ALKBH5) in scars disrupt TGF-β/Smad and Wnt/β-catenin signaling, promoting fibrosis via fibroblast activation, ECM deposition (COL1A1, HIPK2), and YTHDF1-mediated transcript stabilization. Conversely, m^6^A erasers mitigate ECM accumulation through demethylation. METTL3 guides epidermal stem cell differentiation (Wnt/Shh) for hair follicle regeneration, while FTO regulates sweat gland lineage via NANOG. m^6^A further modulates inflammation and necrosis, bridging epitranscriptomic dynamics to fibrotic pathology and tissue repair.

For sweat gland regeneration in superficial wounds, basal cells in the sweat ducts proliferate and differentiate to restore sweat gland structure, but in severe wounds, sweat glands fail to regenerate 136. Autologous pluripotent stem cells and sweat gland-derived progenitor cells, with their strong regenerative abilities and excellent compatibility, hold great potential for regenerating sweat glands in large wounds 137. For instance, TNF-α decreased the expression of the m^6^A writer FTO, which increased the m^6^A level of NANOG mRNA in the 3D culture of MSCs 138. This led to a decrease in NANOG mRNA and protein expression, thereby inhibiting the differentiation of MSCs into sweat gland cells. The demethylation activity of FTO activated the differentiation potential of MSCs, while the TNF-α-FTO axis modified the level of m^6^A modification to regulate the differentiation of MSCs, thus providing an improved idea of stem cell therapies for sweat gland regeneration** (Figure 4)**.

## Discussion

The process of skin wound formation and post-wound events includes the causes of wound formation, the physiological and pathological mechanisms involved in wound healing, and the ultimate outcomes, encompassing the entire spectrum from wound initiation to complete healing 139-141. Among these processes, the role of epigenetic regulation in cellular regulation is of paramount importance. The previous epigenetic studies on skin wound healing have primarily focused on ncRNAs, investigating regulatory mechanisms of chromatin and mRNA through miRNAs and lncRNAs 142,143. Additionally, epigenetic modifications of RNA methylation such as m^1^A, m^5^C, m^7^G, and m^3^C have been identified and implicated in general RNA metabolism, their specific roles in wound healing and scar formation remain largely unexplored 144. In contrast, m^6^A has emerged as the most extensively studied and functionally validated RNA modification, with robust evidence supporting its involvement in altering RNA splicing, half-life, and stability, thereby influencing transcription and translation processes 145. This modification is implicated in numerous stages of skin wound healing and post-wound events, including skin development, cellular migration, differentiation, proliferation, environmental responses, inflammatory reactions, and autophagy 146.

While murine and *in vitro* studies provide foundational insights into m^6^A dynamics, the interspecies differences necessitate cautious extrapolation to human pathophysiology. Murine wound healing exhibits accelerated re-epithelialization and reduced fibrosis compared to humans, potentially underestimating pathological scarring or chronic inflammation in diabetic or aged microenvironments 26. Although it is indispensable to dissect core molecular pathways, conventional *in vitro* systems intrinsically lack the intercellular connections, real physiological context, and key immune factors that define the dynamic human wounding environment, consequently failing to demonstrate complete pathophysiological processes. 20. Future studies should prioritize human tissue spatial transcriptomics or skin organoids to validate clinically translatable m^6^A targets.

The extent of research on the clinical applications of m^6^A remains considerably limited compared to studies investigating its mechanisms, with most studies focusing on its role as a therapeutic target and biomarker in cancer 152. Diagnostic models incorporating m^6^A-related regulators for tumor diagnosis and prognosis evaluation have been established through the analysis of RNA sequencing data 153,154. In addition, the level of tumor-associated m^6^A regulators mediates gene expression regulation, which can enhance drug sensitivity and mitigate resistance, thereby improving the efficacy of immunotherapy, radiotherapy, and chemotherapy 155,156. However, these advances remain insufficient for clinical translation in wound management. To bridge this gap, it's necessary for future research to establish a comprehensive profiling of spatiotemporal m^6^A dynamics across healing phases, enabling temporally precise interventions. And identifying disease-critical RNA molecules through m^6^A reader-writer-eraser networks will empower targeted epitranscriptome editing for precision therapeutics. Critically, overcoming current interspecies limitations requires transitioning to human-relevant validation platforms, which replicate pathophysiological microenvironments to evaluate therapeutic efficacy before clinical translation. In parallel, in wound formation and post-wound events, studies primarily focus on sequencing and mechanisms, with clinical applications remaining unexplored. Although Yang et al. and Xie et al. constructed diagnostic models for keloid formation based on m^6^A-related genes, these models lack reliability due to small sample sizes and insufficient validation 120,121. Current research predominantly leverages m^6^A regulators to modulate target expression, underscoring the imperative to translate epitranscriptomic modifications into clinical therapeutics.

## Conclusion

The emerging field of epitranscriptomics has significantly advanced our understanding of m^6^A RNA methylation as a crucial regulator in skin wound healing and post-wound events. This review highlights the dual role of m^6^A modifications in both physiological repair and pathological conditions such as diabetic ulcers, burns, and HSs. Key regulators, including METTL3, IGF2BP2, and ALKBH5, are pivotal in modulating RNA stability and cellular processes. Despite progress, challenges remain, including fragmented research and limited clinical applications. Resolving the spatiotemporal dynamics of m^6^A methylation during healing transitions and the cell-specificity of reader functions demands integrated single-cell and spatial epigenomic technologies. Future studies should also focus on integrating temporal m^6^A profiling and multi-omics approaches, aiming to develop targeted therapies that improve chronic wound healing and restore skin homeostasis.

## Figures and Tables

**Table 1:** Summary of key m^6^A regulators and their roles in wound healing and post-wound events

Type	Regulator	Biological process	Target	Sample	Biological function	References
**m^6^A writers**	METTL3	Diabetic wound	lncCCKAR-5	hUCMSCs, mice	METTL3-mediated m^6^A modification of lncCCKAR-5 enhances the formation of the lncCCKAR5/LMNA/MKRN2 complex and LMNA degradation in hUCMSCs, thereby inhibiting diabetic wound repair	100
		Keloid	Smad3	HTFs, New Zealand white rabbits	METTL3 promotes TGF-β/Smad signalling by upregulating Smad3, thus enhancing the viability, proliferation, and ECM deposition of HTFs	124
		Hair follicle	Wnt, Shh	Mouse skin epidermal progenitors, C57BL/6 mice	METTL3 ablation in epidermal progenitor cells leads to defects in Wnt and Shh signaling pathways, and reduced HF formation	134
		Hair follicle	SETD1A, SETD1B, KMT2B, KMT2D	C57BL/6 mice	METTL3-eKO newborn mice have abnormal epidermal development and deformed hair follicles.	135
		Keloid	Wnt3a, β-catenin, S100A4	HSFs, Human keloid tissue	Elevated METTL3 and WTAP upregulate the Wnt/β-catenin/S100A4 pathway in human keloid tissue	123
	ZC3H13	Keloid	HIPK2	HKFs, Human keloid tissue	Elevated ZC3H13 mediates the m^6^A modification and stabilisation of HIPK2, thereby promoting HKFs proliferation and accelerating keloid formation	125
	METTL14	Wound	PVT1	Mice	METTL14 modifies lncRNA PVT1 to stabilize MYC, thereby increasing the number of p63-positive basal cells and promoting wound healing	89
**m^6^A readers**	IGF2BP1	Diabetic wound	HMGB1	HUVECs	Increased expression of IGF2BP1 stabilises and increases the expression of HMGB1 mRNA, thereby promoting apoptosis in high glucose-induced HUVECs	147
	IGF2BP2	Wound	HPSE	HaCaT	IGF2BP2 enhances the stability of HPSE, promotes the proliferation and migration of HaCaT cells, and accelerates wound healing	91
		Wound	let-7b	HaCaT, Mice	The overexpression of let-7b inhibits the expression of IGF2BP2 in HaCaT cells, resulting in delayed epithelialisation and skin wound healing	92
	IGF2BP3	Wound	circCDK13	HDFs, HEKs	IGF2BP3 enhances circCDK13 to promote CD44 and c-MYC expression, which enhances the proliferation and migration of HDFs and HEKs and promotes wound healing	105
	YTHDC1	Diabetic wound	SQSTM1	HaCaT, Mice	Downregulation of YTHDC1 in HaCaT cells and mice accelerated SQSTM1 mRNA degradation, leading to delayed diabetic wound healing process	104
**m^6^A erasers**	ALKBH5	Hypertrophic scars	COL1A1, COL3A1, ELN	HDFs, Human HTS tissue	Defects in ALKBH5 lead to elevated m^6^A levels of COL3A1, COL1A1, and ELN, leading to pathological deposition and remodeling of the extracellular matrix	127
	ALKBH5 FTO	Wound	PELI2	HaCaT, Mice	ALKBH5 and YTHDF2 promote PELI2 expression, which promotes keratinocyte migration, epithelialization, and skin wound healing.	97
	FTO	Burn injury	TFPI-2	HaCaT, Rats	Overexpression of FTO inhibits TFPI-2 expression, which promotes the proliferation of thermally stimulated HaCaT cells and accelerates wound healing in burned rats	116
		Keloid	COL1A1, α-SMA	HDFs, Human keloid tissue	Overexpression of FTO in HDFs enhances the expression of COL1A1 and α-SMA, thereby promoting keloid formation	126
		Sweat gland	Nanog	MSCs	Inhibition of FTO expression by TNF-α leads to down-regulation of Nanog expression as well as a reduced potential for MSCs to differentiate into sweat glands.	138

## References

[1] Tobin DJ (2006). Biochemistry of human skin-our brain on the outside. Chem Soc Rev.

[2] Bouwstra JA, Nădăban A, Bras W, McCabe C, Bunge A, Gooris GS (2023). The skin barrier: An extraordinary interface with an exceptional lipid organization. Prog Lipid Res.

[3] Sen CK (2021). Human Wound and Its Burden: Updated 2020 Compendium of Estimates. Adv Wound Care.

[4] Nussbaum SR, Carter MJ, Fife CE (2018). An Economic Evaluation of the Impact, Cost, and Medicare Policy Implications of Chronic Nonhealing Wounds. Value Health J Int Soc Pharmacoeconomics Outcomes Res.

[5] Robles DT, Berg D (2007). Abnormal wound healing: keloids. Clin Dermatol.

[6] Walsh LA, Wu E, Pontes D (2023). Keloid treatments: an evidence-based systematic review of recent advances. Syst Rev.

[7] Rodrigues M, Kosaric N, Bonham CA, Gurtner GC (2019). Wound Healing: A Cellular Perspective. Physiol Rev.

[8] Godo S, Shimokawa H (2017). Endothelial Functions. Arterioscler Thromb Vasc Biol.

[9] Furie B, Furie BC (2008). Mechanisms of thrombus formation. N Engl J Med.

[10] de Bont CM, Boelens WC, Pruijn GJM (2019). NETosis, complement, and coagulation: a triangular relationship. Cell Mol Immunol.

[11] Gurtner GC, Werner S, Barrandon Y, Longaker MT (2008). Wound repair and regeneration. Nature.

[12] Galli SJ, Borregaard N, Wynn TA (2011). Phenotypic and functional plasticity of cells of innate immunity: macrophages, mast cells and neutrophils. Nat Immunol.

[13] Van Dyken SJ, Locksley RM (2013). Interleukin-4- and interleukin-13-mediated alternatively activated macrophages: roles in homeostasis and disease. Annu Rev Immunol.

[14] Stein M, Keshav S, Harris N, Gordon S (1992). Interleukin 4 potently enhances murine macrophage mannose receptor activity: a marker of alternative immunologic macrophage activation. J Exp Med.

[15] Peña OA, Martin P (2024). Cellular and molecular mechanisms of skin wound healing. Nat Rev Mol Cell Biol.

[16] Martin P (1997). Wound healing-aiming for perfect skin regeneration. Science.

[17] Zhu Z, Zhou S, Li S, Gong S, Zhang Q (2024). Neutrophil extracellular traps in wound healing. Trends Pharmacol Sci.

[18] Chu JY, McCormick B, Vermeren S (2018). Small GTPase-dependent regulation of leukocyte-endothelial interactions in inflammation. Biochem Soc Trans.

[19] Hinz B, Gabbiani G (2003). Cell-matrix and cell-cell contacts of myofibroblasts: role in connective tissue remodeling. Thromb Haemost.

[20] Xiaojie W, Banda J, Qi H (2022). Scarless wound healing: Current insights from the perspectives of TGF-β, KGF-1, and KGF-2. Cytokine Growth Factor Rev.

[21] Powers JG, Higham C, Broussard K, Phillips TJ (2016). Wound healing and treating wounds: Chronic wound care and management. J Am Acad Dermatol.

[22] Matoori S, Veves A, Mooney DJ (2021). Advanced bandages for diabetic wound healing. Sci Transl Med.

[23] Krzyszczyk P, Schloss R, Palmer A, Berthiaume F (2018). The Role of Macrophages in Acute and Chronic Wound Healing and Interventions to Promote Pro-wound Healing Phenotypes. Front Physiol.

[24] Theocharidis G, Veves A (2020). Autonomic nerve dysfunction and impaired diabetic wound healing: The role of neuropeptides. Auton Neurosci Basic Clin.

[25] Tellechea A, Bai S, Dangwal S (2020). Topical Application of a Mast Cell Stabilizer Improves Impaired Diabetic Wound Healing. J Invest Dermatol.

[26] Eming SA, Martin P, Tomic-Canic M (2014). Wound repair and regeneration: mechanisms, signaling, and translation. Sci Transl Med.

[27] Tolles J (2018). Emergency department management of patients with thermal burns. Emerg Med Pract.

[28] Hettiaratchy S, Dziewulski P (2004). ABC of burns: pathophysiology and types of burns. BMJ.

[29] Kowalske KJ (2011). Burn wound care. Phys Med Rehabil Clin N Am.

[30] Rowan MP, Cancio LC, Elster EA (2015). Burn wound healing and treatment: review and advancements. Crit Care Lond Engl.

[31] Derkenne C, Ronchi L, Prunet B (2019). Management of Burns. N Engl J Med.

[32] Rae L, Fidler P, Gibran N (2016). The Physiologic Basis of Burn Shock and the Need for Aggressive Fluid Resuscitation. Crit Care Clin.

[33] Sood RF, Gibran NS, Arnoldo BD (2016). Early leukocyte gene expression associated with age, burn size, and inhalation injury in severely burned adults. J Trauma Acute Care Surg.

[34] Ekstein SF, Wyles SP, Moran SL, Meves A (2021). Keloids: a review of therapeutic management. Int J Dermatol.

[35] Ogawa R (2017). Keloid and Hypertrophic Scars Are the Result of Chronic Inflammation in the Reticular Dermis. Int J Mol Sci.

[36] Pakshir P, Noskovicova N, Lodyga M (2020). The myofibroblast at a glance. J Cell Sci.

[37] Kidzeru EB, Lebeko M, Sharma JR (2023). Immune cells and associated molecular markers in dermal fibrosis with focus on raised cutaneous scars. Exp Dermatol.

[38] Syed F, Ahmadi E, Iqbal SA, Singh S, McGrouther DA, Bayat A (2011). Fibroblasts from the growing margin of keloid scars produce higher levels of collagen I and III compared with intralesional and extralesional sites: clinical implications for lesional site-directed therapy. Br J Dermatol.

[39] Niemann C, Horsley V (2012). Development and homeostasis of the sebaceous gland. Semin Cell Dev Biol.

[40] Schneider MR, Schmidt-Ullrich R, Paus R (2009). The hair follicle as a dynamic miniorgan. Curr Biol CB.

[41] Ito M, Liu Y, Yang Z (2005). Stem cells in the hair follicle bulge contribute to wound repair but not to homeostasis of the epidermis. Nat Med.

[42] Rittié L, Sachs DL, Orringer JS, Voorhees JJ, Fisher GJ (2013). Eccrine sweat glands are major contributors to reepithelialization of human wounds. Am J Pathol.

[43] Jiang X, Liu B, Nie Z (2021). The role of m6A modification in the biological functions and diseases. Signal Transduct Target Ther.

[44] Ling C, Rönn T (2019). Epigenetics in Human Obesity and Type 2 Diabetes. Cell Metab.

[45] Jones PA, Issa J-PJ, Baylin S (2016). Targeting the cancer epigenome for therapy. Nat Rev Genet.

[46] Li Y, Jin H, Li Q, Shi L, Mao Y, Zhao L (2024). The role of RNA methylation in tumor immunity and its potential in immunotherapy. Mol Cancer.

[47] Lan Q, Liu PY, Haase J, Bell JL, Hüttelmaier S, Liu T (2019). The Critical Role of RNA m6A Methylation in Cancer. Cancer Res.

[48] Chen Y, Jiang Z, Yang Y, Zhang C, Liu H, Wan J (2023). The functions and mechanisms of post-translational modification in protein regulators of RNA methylation: Current status and future perspectives. Int J Biol Macromol.

[49] Wei CM, Gershowitz A, Moss B (1975). Methylated nucleotides block 5' terminus of HeLa cell messenger RNA. Cell.

[50] Fu Y, Dominissini D, Rechavi G, He C (2014). Gene expression regulation mediated through reversible m^6^A RNA methylation. Nat Rev Genet.

[51] Rottman F, Shatkin AJ, Perry RP (1974). Sequences containing methylated nucleotides at the 5' termini of messenger RNAs: possible implications for processing. Cell.

[52] Liu J, Yue Y, Han D (2014). A METTL3-METTL14 complex mediates mammalian nuclear RNA N6-adenosine methylation. Nat Chem Biol.

[53] Zheng G, Dahl JA, Niu Y (2013). ALKBH5 is a mammalian RNA demethylase that impacts RNA metabolism and mouse fertility. Mol Cell.

[54] Jia G, Fu Y, Zhao X (2011). N6-methyladenosine in nuclear RNA is a major substrate of the obesity-associated FTO. Nat Chem Biol.

[55] Dominissini D, Moshitch-Moshkovitz S, Schwartz S (2012). Topology of the human and mouse m6A RNA methylomes revealed by m6A-seq. Nature.

[56] Meyer KD, Saletore Y, Zumbo P, Elemento O, Mason CE, Jaffrey SR (2012). Comprehensive analysis of mRNA methylation reveals enrichment in 3' UTRs and near stop codons. Cell.

[57] Cerneckis J, Ming G-L, Song H, He C, Shi Y (2024). The rise of epitranscriptomics: recent developments and future directions. Trends Pharmacol Sci.

[58] Acera Mateos P, Zhou Y, Zarnack K, Eyras E (2023). Concepts and methods for transcriptome-wide prediction of chemical messenger RNA modifications with machine learning. Brief Bioinform.

[59] Hess ME, Hess S, Meyer KD (2013). The fat mass and obesity associated gene (Fto) regulates activity of the dopaminergic midbrain circuitry. Nat Neurosci.

[60] Hart SM, Foroni L (2002). Core binding factor genes and human leukemia. Haematologica.

[61] Ju C-C, Liu X-X, Liu L (2024). Epigenetic modification: A novel insight into diabetic wound healing. Heliyon.

[62] Feng Z, Li Q, Meng R, Yi B, Xu Q (2018). METTL3 regulates alternative splicing of MyD88 upon the lipopolysaccharide-induced inflammatory response in human dental pulp cells. J Cell Mol Med.

[63] Wang J, Yan S, Lu H, Wang S, Xu D (2019). METTL3 Attenuates LPS-Induced Inflammatory Response in Macrophages via NF-κB Signaling Pathway. Mediators Inflamm.

[64] Zhang Y, Gu X, Li D, Cai L, Xu Q (2020). METTL3 Regulates Osteoblast Differentiation and Inflammatory Response via Smad Signaling and MAPK Signaling. Int J Mol Sci.

[65] Huangfu N, Zheng W, Xu Z (2020). RBM4 regulates M1 macrophages polarization through targeting STAT1-mediated glycolysis. Int Immunopharmacol.

[66] Liu Y, Liu Z, Tang H (2019). The N6-methyladenosine (m6A)-forming enzyme METTL3 facilitates M1 macrophage polarization through the methylation of STAT1 mRNA. Am J Physiol-Cell Physiol.

[67] Yan C, Xiong J, Zhou Z (2023). A cleaved METTL3 potentiates the METTL3-WTAP interaction and breast cancer progression. eLife.

[68] Hu J, Lin H, Wang C, Su Q, Cao B (2023). METTL14-mediated RNA methylation in digestive system tumors. Int J Mol Med.

[69] Mansfield KD (2024). RNA Binding by the m6A Methyltransferases METTL16 and METTL3. Biology.

[70] Dou X, Huang L, Xiao Y (2023). METTL14 is a chromatin regulator independent of its RNA N6-methyladenosine methyltransferase activity. Protein Cell.

[71] Truffinet F, Arco-Hierves A, Shalabi H m6A RNA methylation controls salivary gland epithelial cell function and has a protective role in Sjögren's disease. Ann Rheum Dis. 2024: ard-2024-226224.

[72] Barone S, Cerchia C, Summa V, Brindisi M (2024). Methyl-Transferase-Like Protein 16 (METTL16): The Intriguing Journey of a Key Epitranscriptomic Player Becoming an Emerging Biological Target. J Med Chem.

[73] Wei G (2024). RNA m6A modification, signals for degradation or stabilisation?. Biochem Soc Trans.

[74] Chen L, Gao Y, Xu S (2023). N6-methyladenosine reader YTHDF family in biological processes: Structures, roles, and mechanisms. Front Immunol.

[75] Chen D, Cheung H, Lau HC-H, Yu J, Wong CC (2022). N6-Methyladenosine RNA-Binding Protein YTHDF1 in Gastrointestinal Cancers: Function, Molecular Mechanism and Clinical Implication. Cancers.

[76] Kaur S, Tam NY, McDonough MA, Schofield CJ, Aik WS (2022). Mechanisms of substrate recognition and N6-methyladenosine demethylation revealed by crystal structures of ALKBH5-RNA complexes. Nucleic Acids Res.

[77] Lai G-Q, Li Y, Zhu H (2024). A covalent compound selectively inhibits RNA demethylase ALKBH5 rather than FTO. RSC Chem Biol.

[78] Wei J, Yu X, Yang L (2022). FTO mediates LINE1 m6A demethylation and chromatin regulation in mESCs and mouse development. Science.

[79] Huang Y, Xia W, Dong Z, Yang C-G (2023). Chemical Inhibitors Targeting the Oncogenic m6A Modifying Proteins. Acc Chem Res.

[80] Li W, Zhou J, Gu Y (2024). Lactylation of RNA m6A demethylase ALKBH5 promotes innate immune response to DNA herpesviruses and mpox virus. Proc Natl Acad Sci U S A.

[81] Zuidhof HR, Calkhoven CF (2022). Oncogenic and Tumor-Suppressive Functions of the RNA Demethylase FTO. Cancer Res.

[82] Yu M, Ji W, Yang X (2023). The role of m6A demethylases in lung cancer: diagnostic and therapeutic implications. Front Immunol.

[83] Azzam SK, Alsafar H, Sajini AA (2022). FTO m6A Demethylase in Obesity and Cancer: Implications and Underlying Molecular Mechanisms. Int J Mol Sci.

[84] Kopp F, Mendell JT (2018). Functional Classification and Experimental Dissection of Long Noncoding RNAs. Cell.

[85] Derrien T, Johnson R, Bussotti G (2012). The GENCODE v7 catalog of human long noncoding RNAs: analysis of their gene structure, evolution, and expression. Genome Res.

[86] Jin Y, Fan Z (2024). New insights into the interaction between m6A modification and lncRNA in cancer drug resistance. Cell Prolif.

[87] Yue Y, Liu J, He C (2015). RNA N6-methyladenosine methylation in post-transcriptional gene expression regulation. Genes Dev.

[88] Tseng Y-Y, Moriarity BS, Gong W (2014). PVT1 dependence in cancer with MYC copy-number increase. Nature.

[89] Lee J, Wu Y, Harada BT (2021). N^6^-methyladenosine modification of lncRNA *Pvt1* governs epidermal stemness. EMBO J.

[90] Huang H, Weng H, Sun W (2018). Recognition of RNA N6-methyladenosine by IGF2BP proteins enhances mRNA stability and translation. Nat Cell Biol.

[91] Zhi S, Li J, Kong X, Xie X, Zhang Q, Fang G (2021). Insulin-like growth factor 2 mRNA binding protein 2 regulates proliferation, migration, and angiogenesis of keratinocytes by modulating heparanase stability. Bioengineered.

[92] Wu Y, Zhong JL, Hou N (2017). MicroRNA Let-7b inhibits keratinocyte migration in cutaneous wound healing by targeting IGF2BP2. Exp Dermatol.

[93] Gao Z, Zha X, Li M, Xia X, Wang S (2024). Insights into the m6A demethylases FTO and ALKBH5 : structural, biological function, and inhibitor development. Cell Biosci.

[94] Wang D, Zheng T, Zhou S (2023). Promoting axon regeneration by inhibiting RNA N6-methyladenosine demethylase ALKBH5. Gleeson JG, Bronner ME, Yoon K-J, Wolozin B, Eds. eLife.

[95] J Z, X Z, J H (2021). m6A demethylase ALKBH5 controls CD4+ T cell pathogenicity and promotes autoimmunity. Sci Adv.

[96] Liu Y, Song R, Zhao L (2022). m6A demethylase ALKBH5 is required for antibacterial innate defense by intrinsic motivation of neutrophil migration. Signal Transduct Target Ther.

[97] Huang X, Zhao Y, Liu D (2023). ALKBH5-mediated m6A demethylation fuels cutaneous wound re-epithelialization by enhancing PELI2 mRNA stability. Inflamm Regen.

[98] Shen J, Chen H, Dai J (2024). Genome-wide screening of m6A profiling of cutaneous wound healing in diabetic mice. Mol Biol Rep.

[99] Yan C, Xv Y, Lin Z (2022). Human Umbilical Cord Mesenchymal Stem Cell-Derived Exosomes Accelerate Diabetic Wound Healing via Ameliorating Oxidative Stress and Promoting Angiogenesis. Front Bioeng Biotechnol.

[100] Wang J, Wang X, Chen F (2024). N6-Methyladenosine Modification of lncCCKAR-5 Regulates Autophagy in Human Umbilical Cord Mesenchymal Stem Cells by Destabilizing LMNA and Inhibits Diabetic Wound Healing. J Invest Dermatol.

[101] Li L, Zhang J, Zhang Q (2019). High Glucose Suppresses Keratinocyte Migration Through the Inhibition of p38 MAPK/Autophagy Pathway. Front Physiol.

[102] Chen J, Wang C, Fei W, Fang X, Hu X (2019). Epitranscriptomic m6A modification in the stem cell field and its effects on cell death and survival. Am J Cancer Res.

[103] Yan H, Zhang L, Cui X, Zheng S, Li R (2022). Roles and mechanisms of the m6A reader YTHDC1 in biological processes and diseases. Cell Death Discov.

[104] Liang D, Lin W-J, Ren M (2022). m6A reader YTHDC1 modulates autophagy by targeting SQSTM1 in diabetic skin. Autophagy.

[105] Huang Q, Chu Z, Wang Z (2024). circCDK13-loaded small extracellular vesicles accelerate healing in preclinical diabetic wound models. Nat Commun.

[106] Wang T, Li X, Tao Y, Wang X, Li L, Liu J (2024). METTL3-mediated NDUFB5 m6A modification promotes cell migration and mitochondrial respiration to promote the wound healing of diabetic foot ulcer. J Transl Med.

[107] Zhou J, Wei T, He Z (2021). ADSCs enhance VEGFR3-mediated lymphangiogenesis via METTL3-mediated VEGF-C m6A modification to improve wound healing of diabetic foot ulcers. Mol Med.

[108] Jeschke MG, Chinkes DL, Finnerty CC (2008). Pathophysiologic response to severe burn injury. Ann Surg.

[109] Guillory AN, Clayton RP, Herndon DN, Finnerty CC (2016). Cardiovascular Dysfunction Following Burn Injury: What We Have Learned from Rat and Mouse Models. Int J Mol Sci.

[110] Jeschke MG, van Baar ME, Choudhry MA, Chung KK, Gibran NS, Logsetty S (2020). Burn injury. Nat Rev Dis Primer.

[111] Ran Y, Yan Z, Huang M (2023). Severe Burn Injury Significantly Alters the Gene Expression and m6A Methylation Tagging of mRNAs and lncRNAs in Human Skin. J Pers Med.

[112] Zhang L, Wan Y, Zhang Z (2021). FTO demethylates m6A modifications in HOXB13 mRNA and promotes endometrial cancer metastasis by activating the WNT signalling pathway. RNA Biol.

[113] Xu Y, Ye S, Zhang N (2020). The FTO/miR-181b-3p/ARL5B signaling pathway regulates cell migration and invasion in breast cancer. Cancer Commun Lond Engl.

[114] Kang H, Zhang Z, Yu L, Li Y, Liang M, Zhou L (2018). FTO reduces mitochondria and promotes hepatic fat accumulation through RNA demethylation. J Cell Biochem.

[115] Cui Y-H, Yang S, Wei J (2021). Autophagy of the m6A mRNA demethylase FTO is impaired by low-level arsenic exposure to promote tumorigenesis. Nat Commun.

[116] Xu Z, Zhu X, Mu S (2024). FTO overexpression expedites wound healing and alleviates depression in burn rats through facilitating keratinocyte migration and angiogenesis via mediating TFPI-2 demethylation. Mol Cell Biochem.

[117] Bayat A, McGrouther DA, Ferguson MWJ (2003). Skin scarring. BMJ.

[118] Jeschke MG, Wood FM, Middelkoop E (2023). Scars. Nat Rev Dis Primer.

[119] Gauglitz GG, Korting HC, Pavicic T, Ruzicka T, Jeschke MG (2011). Hypertrophic Scarring and Keloids: Pathomechanisms and Current and Emerging Treatment Strategies. Mol Med.

[120] Yang R, Wang X, Zheng W (2023). Bioinformatics analysis and verification of m6A related genes based on the construction of keloid diagnostic model. Int Wound J.

[121] Xie J, Zhang X, Zhang K (2023). Construction and validation of the diagnostic model of keloid based on weighted gene co-expression network analysis (WGCNA) and differential expression analysis. J Plast Surg Hand Surg.

[122] Liu S-Y, Wu J-J, Chen Z (2021). The m6A RNA Modification Modulates Gene Expression and Fibrosis-Related Pathways in Hypertrophic Scar. Front Cell Dev Biol.

[123] Lin C-X, Chen Z-J, Peng Q-L (2022). The m6A-methylated mRNA pattern and the activation of the Wnt signaling pathway under the hyper-m6A-modifying condition in the keloid. Front Cell Dev Biol.

[124] Liu Y, Gu C, Li X, Wang T, Yu L (2021). Involvement of METTL3/m6Adenosine and TGFβ/Smad3 signaling on Tenon's fibroblasts and in a rabbit model of glaucoma surgery. J Mol Histol.

[125] Fu M, Chen Y, Shi X (2024). ZC3H13 Accelerates Keloid Formation by Mediating N6-methyladenosine Modification of HIPK2. Biochem Genet.

[126] Ren S, Ji Y, Wang M, Ye M, Huang L, Cai X (2023). The m6A demethylase FTO promotes keloid formation by up-regulating COL1A1. Ann Transl Med.

[127] Xu R, Yang E, Liang H (2024). ALKBH5-mediated m6A demethylation ameliorates extracellular matrix deposition in cutaneous pathological fibrosis. Clin Transl Med.

[128] Takeo M, Lee W, Ito M (2015). Wound Healing and Skin Regeneration. Cold Spring Harb Perspect Med.

[129] Batista PJ, Molinie B, Wang J (2014). m6A RNA Modification Controls Cell Fate Transition in Mammalian Embryonic Stem Cells. Cell Stem Cell.

[130] Geula S, Moshitch-Moshkovitz S, Dominissini D (2015). m6A mRNA methylation facilitates resolution of naïve pluripotency toward differentiation. Science.

[131] Rezza A, Wang Z, Sennett R (2016). Signaling Networks among Stem Cell Precursors, Transit-Amplifying Progenitors, and their Niche in Developing Hair Follicles. Cell Rep.

[132] Rishikaysh P, Dev K, Diaz D, Qureshi WMS, Filip S, Mokry J (2014). Signaling Involved in Hair Follicle Morphogenesis and Development. Int J Mol Sci.

[133] Oak ASW, Cotsarelis G (2023). Wound-Induced Hair Neogenesis: A Portal to the Development of New Therapies for Hair Loss and Wound Regeneration. Cold Spring Harb Perspect Biol.

[134] Xi L, Carroll T, Matos I (2020). m6A RNA methylation impacts fate choices during skin morphogenesis. Horsley V, Bronner ME, Yi R, Eds. eLife.

[135] Maldonado López AM, Ko EK, Huang S (2023). Mettl3-catalyzed m6A regulates histone modifier and modification expression in self-renewing somatic tissue. Sci Adv.

[136] Lobitz WC, Holyoke JB, Montagna W (1954). Responses of the Human Eccrine Sweat Duct to Controlled Injury: Growth Center of the “Epidermal Sweat Duct Unit”1. J Invest Dermatol.

[137] Chen R, Zhu Z, Ji S (2020). Sweat gland regeneration: Current strategies and future opportunities. Biomaterials.

[138] Wang Y, Wang R, Yao B (2020). TNF-α suppresses sweat gland differentiation of MSCs by reducing FTO-mediated m6A-demethylation of Nanog mRNA. Sci China Life Sci.

[139] Martin P, Nunan R (2015). Cellular and molecular mechanisms of repair in acute and chronic wound healing. Br J Dermatol.

[140] den Dekker A, Davis FM, Kunkel SL, Gallagher KA (2019). Targeting epigenetic mechanisms in diabetic wound healing. Transl Res J Lab Clin Med.

[141] Chen C, Liu T, Tang Y, Luo G, Liang G, He W (2023). Epigenetic regulation of macrophage polarization in wound healing. Burns Trauma.

[142] Herter EK, Xu Landén N (2017). Non-Coding RNAs: New Players in Skin Wound Healing. Adv Wound Care.

[143] Ozdemir D, Feinberg MW (2019). MicroRNAs in diabetic wound healing: Pathophysiology and therapeutic opportunities. Trends Cardiovasc Med.

[144] Barbieri I, Kouzarides T (2020). Role of RNA modifications in cancer. Nat Rev Cancer.

[145] Wang X, Lu Z, Gomez A (2014). N6-methyladenosine-dependent regulation of messenger RNA stability. Nature.

[146] Kiya K, Kubo T (2019). Neurovascular interactions in skin wound healing. Neurochem Int.

[147] Liang A, Liu J, Wei Y (2023). m6A reader IGF2BP1 accelerates apoptosis of high glucose-induced vascular endothelial cells in a m6A-HMGB1 dependent manner. PeerJ.

[148] Linder B, Grozhik AV, Olarerin-George AO, Meydan C, Mason CE, Jaffrey SR (2015). Single-nucleotide-resolution mapping of m6A and m6Am throughout the transcriptome. Nat Methods.

[149] Shi H, Wei J, He C (2019). Where, When, and How: Context-Dependent Functions of RNA Methylation Writers, Readers, and Erasers. Mol Cell.

[150] Qiu L, Jing Q, Li Y, Han J (2023). RNA modification: mechanisms and therapeutic targets. Mol Biomed.

[151] Zhang H, Shi X, Huang T (2020). Dynamic landscape and evolution of m6A methylation in human. Nucleic Acids Res.

[152] An Y, Duan H (2022). The role of m6A RNA methylation in cancer metabolism. Mol Cancer.

[153] Gu Y, Wu X, Zhang J (2021). The evolving landscape of N6-methyladenosine modification in the tumor microenvironment. Mol Ther J Am Soc Gene Ther.

[154] Huang J, Yang J, Zhang Y, Lu D, Dai Y (2023). FTO promotes cervical cancer cell proliferation, colony formation, migration and invasion via the regulation of the BMP4/Hippo/YAP1/TAZ pathway. Exp Cell Res.

[155] Wang L, Hui H, Agrawal K (2020). m6 A RNA methyltransferases METTL3/14 regulate immune responses to anti-PD-1 therapy. EMBO J.

[156] Yan F, Al-Kali A, Zhang Z (2018). A dynamic N6-methyladenosine methylome regulates intrinsic and acquired resistance to tyrosine kinase inhibitors. Cell Res.

